# Prevalence, serovars, and risk factors associated with the presence of *Salmonella* in pork sold in public markets in Quito, Ecuador

**DOI:** 10.12688/f1000research.138671.2

**Published:** 2024-04-17

**Authors:** Christian Vinueza-Burgos, Luis Hidalgo-Arellano, Carlos Gómez-Coronado, José Luis Medina-Santana, María Cevallos-Almeida

**Affiliations:** 1 Unidad de Investigación de Enfermedades Transmitidas por Alimentos y Resistencia a los Antimicrobianos (UNIETAR). Facultad de Medicina Veterinaria y Zootecnia, Universidad Central del Ecuador, Quito, Pichincha, Ecuador; 2 Laboratorio de Bacteriología y Micología, Facultad de Medicina Veterinaria y Zootecnia, Universidad Central del Ecuador, Quito, Pichincha, Ecuador

**Keywords:** Salmonella, Infantis, monophasic S. Typhimurium, Derby, risk factors, pork, antibiotic resistance, Ecuador, Beta-lactamase

## Abstract

**Background:**

*Salmonella enterica* are bacteria that include more than 2,500 serovars. Most of these serovars have been linked to human foodborne illnesses, mainly related to poultry and pigs. Thus, these animals are considered the reservoirs of many *Salmonella* serovars and strains related to antibiotic resistance. This study aimed to determine the prevalence, serovars, β-lactam resistance genes, and the risk factors associated with *Salmonella enterica* in pork commercialized in open markets of Quito city.

**Methods:**

For this, 165 pork meat samples were taken from municipal markets in three areas in the city. These samples were microbiologically processed following the ISO 6579-2014 standardized method. The polymerase chain reaction (PCR) test was used to identify *Salmonella* serotyping and resistance genes. Strains not identified by PCR were typed by the Kauffman White Le Minor scheme. A multivariate analysis was performed to identify risk factors associated with the presence of the microorganism.

**Results:**

*Salmonella* prevalence in pork was 9.1%. Identified serovars were 4, [5], 12: i:- (53.3%), Infantis (33.3%), and Derby (13.4%). Furthermore, the β-lactam resistance genes *bla*_CTX-M-65_ could be identified in three *S. infantis* isolates. Multivariate analysis showed that temperature (above 8°C) and cutting surfaces (wood) presented significant association values.

**Conclusions:**

In conclusion, pork in traditional markets of Quito is contaminated with Salmonella enterica, whose main serovars pose a public health concern, and shows beta-lactam resistance.

## Introduction

The consumption of undercooked food contaminated with non-typhoid *Salmonella* is one of the most important causes of human gastroenteritis worldwide. The World Health Organization (WHO), established that more than 2 billion people worldwide suffer from diarrheal disease annually. ^1^

*Salmonella* transmission to humans happens along the farm-to-fork via contaminated animal- and plant-derived foods, including poultry, eggs, fish, pork, beef, vegetables, fruits, nuts, and flour. ^2^ This contamination can occur at any stage in the production chain. ^3^^,^^4^ Animals such as pigs can be infected or colonized by different *Salmonella* serovars *,* developing a disease or becoming a reservoir of this microorganism, excreting and spreading it. ^5^ Contaminated pork plays an important role as one of the main sources of human infection with *Salmonella* in several countries. ^6^^–^^8^ It has to be considered that cross-contamination of food with *Salmonella* may occur by kitchen instruments, improper hygienic handling, etc. ^9^

Different *Salmonella* serovars can be implicated in a large number of human infections. Therefore, serovar identification is essential in the epidemiological surveillance of this pathogen. ^10^ Several serovars have been reported in pork and pig production, associated with different geographical localization. Thus, *S.* Derby and *S.* Typhimurium were the most prevalent serovars in Europe, Oceania, Asia, and North America, ^11^ while *S.* Derby has been reported as the most pervasive serovar in pork in Latin America. ^12^

Besides, antimicrobial resistance has posed a critical problem for human and animal health in the last 20 years. ^13^ The overuse of antibiotics in these two areas has contributed to the emergence of antibiotic resistance in foodborne bacteria. ^14^ Thus, several studies have reported multi-resistant *Salmonella* strains (MDR) in which *Salmonella* isolates producing extended-spectrum beta-lactamase enzymes (ESBL) are common findings. ^15^^–^^17^ Although the importance of non-typhoid *Salmonella* and its antimicrobial resistance is evident in food production, epidemiological information about the presence of *this* bacteria in pork is still limited in Ecuador. ^18^ Therefore, this research aimed to estimate the prevalence of *Salmonella* serovars, antimicrobial resistance, and risk factors associated with *Salmonella enterica* in pork meat sold in markets in Quito-Ecuador.

## Methods

### Sample collection

Information of total markets in Quito city was provided by the city’s major office report, where 56 local markets are established. According to the geographical distribution of these markets, 18 were selected as follows: 8 in the north of the city, 3 in the center of the city and 7 in the south of the city. The number of samples in every market was assigned according to the size of the market. Sampling and surveys were applied to the butchery employees who agreed to participate in the study. A total of 165 pork samples were collected in 11 traditional markets in three areas of Quito. Samples of 100 g of meat from 25 butcheries were collected between March and May 2021. Each sample was collected in sterile plastic flasks and transported to the laboratory in an icebox at 2–8°C.

### Survey information

An epidemiological questionnaire to estimate the risk factors for pork contamination was developed and applied to the butchery employees Included variables were based in previous studies ^3^^,^^19^ and adapted for this study ( Table 1).

**Table 1.** Variables included in the epidemiological survey.

Variables	Description
Location	North
	Center
	South
Owner's education level	None
	Elemental
	High school
	University
Number of people at the sales stand	One person
	Two people
	>2 people
Functionally age of butchery	<1 year to 5 years
	>5 years
Types of commercialized meats	Only pork
	Two meat products
	>3 meat products
Meat preservation temperature	Temperature < 8°C
	Temperature > 8°C
Meat preservation time	Unknown
	1–2 days
Meat location at the seller’s shop	Metal Hanger/Refrigerator
	Refrigerator/Cold room (CR)
	Refrigerator/CR/freezer
	Metal hangers & trays
Meat cutting surface	Metallic & acrylic
	Wood
Origin of pork	Metropolitan slaughterhouse
	Other origin
Type of water used	Potable Drinking water
	Without water at the butchery
Type of cleaning material	Wipe
	Disposable paper & wipe
Cleaning products used for disinfection	Detergent & degreaser
	>3 Cleaning products

### Isolation and identification of *Salmonella*

*Salmonella* was isolated and identified according to the ISO 6579: 2017 standardized method. ^20^ Briefly, 25 g of pork and 225 ml of Buffered Peptone Water – BPW (BD Difco 218105 – USA) were placed in a sterile zip bag and homogenized to obtain a 1:10 suspension. The combination was incubated at 37°C ± 1°C for 18 h ± 2 h. After incubation, 0.1 ml of the pre-enriched culture as 1–3 equally spaced spots on the surface of Modified Semisolid Rappaport Vassidialis - MSRV plates (BD Difco 218681 – USA) and incubated at 41.5°C for 24 h ± 3 h. A loop of 1 μl was taken inside the edge of the opaque growth in MSRV and plated in the Xylose Lysine, Deoxycholate – XLD agar (BD Difco 278850 – USA) and incubated at 37°C for 24 ± 3 h. Typical colonies of *Salmonella* spp. in XLD (black center with a slightly transparent reddish area) were then identified by biochemical tests. These tests included Triple Sugar Agar – TSI (BD Difco 226540 – USA), Lysine Iron Agar – LIA (BD Difco 284920 – USA), Simmons Citrate BD (Difco 211620 – USA), and Urea broth (BD Difco 227210 – USA). All the positive isolates were conserved at -70°C in a Brain Head Infusion medium (BD Difco 241830 – USA) with glycerol (Fisher Chemical G33500 – USA).

### DNA extraction

DNA extraction was performed using the boiling method. Briefly, *Salmonella* isolates were plated in XLD agar and incubated at 37±1°C for 24 hours. A typical *Salmonella* colony was transferred to a sterile Eppendorf tube containing 300 μl of 1X TE buffer (Tris base Promega H5131 – USA + EDTA Promega V4231 – USA) and lysed at 95°C for 20 minutes. The supernatant was collected and stored at -20°C.

### Serovars identification

Serovars Infantis, Enteritidis, Typhimurium, and its monophasic variant (1,4, [5], 12: i:-) were identified by polymerase chain reaction (PCR), using the *invA* gene as housekeeping control. Primers and annealing temperature for these PCR protocols are described in Table 2. GoTaq ^®^ Flexi DNA Polymerase (Promega M8291 – USA), nuclease-Free Water (Promega P1197 – USA), and dNTP Mix (Promega U1515 – USA) were used as PCR reagents on a SimpliAmp™ Thermal Cycler (Thermo Fisher A24811 – USA) to perform all reactions. According to the Kauffmann-White scheme, *Salmonella* isolates not typed by PCR were serotyped using the agglutination method. ^19^ Briefly, each strain was recovered in Nutrient Agar (BD Difco 213000 – USA) and incubated for 16 to 20 h at 37°C ± 2. Then, the agglutination test was performed, confronting the bacterial suspension to specific antisera in a multi-cup plate. Positive agglutination is visualized by aggregate formation (more or less cottony appearance) exerting a moderate circular agitation of the plaque. Determination of somatic antigen (O antigen) requires the primary test of polyvalent sera (OMA, OMB, OMC, OMD, OME, OMF, and OMG) followed by monovalent ones (Remel™ Agglutinating Sera – UK). To determine flagellar antigen (H antigen), agglutination with one of the mixing sera for orientation (HMA, HMB, HMC) or the versatile serum H1 was observed (Remel™ Agglutinating Sera – UK). The determination of the flagellar antigen is obtained by successive elimination until detection of agglutination with one of the specific sera included in the mixing serum (search for major H then minor H). The combination of somatic antigen and flagellar antigen defines the serotype of the strain under study. The Kauffmann–White scheme gathers the groups and the corresponding sera.

**Table 2.** Primers used for *Salmonella* serovars identification.

Target gene	Primers	Sequence	Amplicons size (pb)	Annealing Tem.	Ref.
*Salmonella* spp.	InvAF	5′-AAACCTAAAACCAGCAAAGG-3′	605	58	^20^
	InvAR	5′-TGTACCGTGGCATGTCTGAG-3′			
*S.* Infantis	IMP1-F	5′-GGTCATTGTCGGAAACCTGC-3′	95	60	^20^
	IMP1-R	5′-ACATTCCCCCTTCCACTGCC-3′			
	IMP2-F	5′-CGCGAAGAAGTGCATAAACC-3′	198	60	
	IMP2-R	5′-CGCCACTTTCGTTATCTGAG-3′			
	IMP3-F	5′-ACCTACTACTATCCCTGATG-3′	304	60	
	IMP3-R	5′-GCGAATTTTGCTACTTGAAG-3′			
*S.* Enteritidis	EMP1-F	5′-AATACAGCCTCAACCAGCTA-3′	101	60	^20^
	EMP1-R	5′-ATTGGTTCACCCGTTGCAAT-3′			
	EMP2-F	5′-AGATAAGCCCTCCCTGCTTA-3	203	60	
	EMP2-R	5′-CCCTCCTTTCACTGCAAGTC-3′			
	EMP3-F	5′-CAAAAGCGACAAATAATCTG-3′	299	60	
	EMP3-R	5′-TTTCTCCGCCTGTTTTCGTT-3′			
*S.* Typhimurium	TMP1-F	5′-ATGCGGGTATGACAAACCCT-3′	94	60	^20^
	TMP1-R	5′-TTAGCCCCATTTGGACCTTT-3′			
	TMP2-F	5′-CAGACCAGGTAAGTTTCTGG-3′	196	60	
	TMP2-R	5′-CGCATATTTGGTGCAGAAAT-3′			
	TMP3-F	5′-TTTACCTCAATGGCGGAACC-3′	303	60	
	TMP3-R	5′-CCCAAAAGCTGGGTTAGCAA-3′			
*S.* Typhimurium Monophasic	MDH F	5′-TGCCAACGGAAGTTGAAGTG-3′	260	58	^21^
	MDH R	5′-CGCATTCCACCACGCCCTTC-3′	550		
	fliC F	5′-ATAGCCATCTTTACCAGTTCC-3′	1389	58	
	fliC R	5′-ACTCAGGCTTCCCGTAACGC-3′			
	fljB F	5′-CAACAACAACCTGCAGCGTGTGCG-3′	964	64	
	fljB R	5′-GCCATATTTCAGCCTCTCGCCCG-3′			
	FFLiB	5′-CTGGCGACGATCTGTCGATG-3′	964	64	
	RFLIA	5′-GCGGTATACAGTGAATTCAC-3′			

### Antimicrobial susceptibility test

Identification of antimicrobial resistance *Salmonella* isolates was carried out using the Kirby-Bauer disk diffusion method according to the Clinical and Laboratory Standards Institute guidelines. ^24^ The clinical cut-offs of M100 CLSI standards (CLSI, 2022) were used as references integrating intermedia values as resistant. The antibiotics used were: Ampicillin (Oxoid, USA; 10 μg), Cefoxitin (Oxoid CT0003B – USA; 30 μg), Cefotaxime (BD 231606 – USA; 30 μg), Ceftazidime (BD 231632 – USA; 30 μg), Amoxicillin + clavulanic acid (Oxoid CT0223B – USA; 30 μg), Ertapenem (BD 232175 – USA 10 μg), Tetracycline (Oxoid CT0054B – USA; 30 μg), Tigecycline (Oxoid CT1841B – USA; 15 μg), Chloramphenicol (Oxoid CT0013B – USA; 30 μg), Ciprofloxacin (Oxoid CT0425B – USA; 5 μg), Sulfamethoxazole + Trimethoprim (Oxoid CT0052B – USA; 30 μg), Gentamicin (Oxoid CT0024B – USA; 10 μg), Amikacin (Oxoid CT0107B – USA; 30 μg), Nitrofurantoin (Oxoid CT0036B – USA; 300 μg), Fosfomycin (Oxoid CT0758B – USA; 200 μg). The *E. coli* ATCC 25922 was used as control strain.

PCR further studied isolates with phenotypic resistance to beta-lactams to detect *bla*_CTX-M_, *bla*_TEM_, *bla*_CMY_, and *bla*_SHV_ genes. Additionally, the *bla*_CTX-M_ subgroups were identified for later sequencing. Primers and annealing temperature for these PCR protocols are described in Table 3. PCR reagents and equipment used for serotyping by PCR were used in this step.

**Table 3.** Primers used for Beta-lactams identification.

Target gene	Primers	Sequence	Amplicon size (pb)	Annealing Tem.	Ref.
*bla*_CTX-M General_	CTX-MU1	5′-ATG TGC AGY ACC AGT AAR GTK ATG GC	592	60	^25^^,^^26^
	CTX-MU2	5′-TGG GTR AAR TAR GTS ACC AGA AYS AGC GG			
*bla*_SHV_	SHVOS5	5′-TTA TCT CCC TGT TAG CCA CC	795	60	^26^^,^^27^
	SHVOS6	5′-GAT TTG CTG ATT TCG CTC GG			
*bla*_TEM_	TEM front P1	5′-GCG GAA CCC CTA TTT G	964	55	^26^^,^^28^
	TEM-C-R-ny	5′-ACC AAT GCT TAA TCA GTG AG			
*bla*_CMY_	Queprev cmy-2 sart	5′-ATG ATG AAA AAA TCG TTA TGC TGC	1117	60	^25^^,^^26^
	cmy-group2-R	5′-GCT TTT CAA GAA TGC GCC AGG			
*bla*_CTX-M1 Group_	CTX-1-SEQ-F	5′-CCC ATG GTT AAA AAA TCA CTG C	1000	60	^29^^,^^30^
	CTX-1-SEQ-R	5′-CAG CGC TTT TGC CGT CTA AG			
*bla*_CTX-M2 Group_	CTX-M-2F	5′-ATG ATG ACT CAG AGC ATT CG	1179	60	^31^^,^^32^
	CTX-M-2R	5′-TGG GTT ACG ATT TTC GCC GC			
*bla*_CTX-M8 Group_	CTX-Mgp8-F	5′-TGA TGA GAC ATC GCG TTA AG	871	55	^33^^,^^34^
	CTX-Mgp8-R	5′-TAA CCG TCG GTG ACG ATT TT			
*bla*_CTX-M9 Group_	CTX-M-9-1F	5′-TGG TGA CAA AGA GAG TGC AAC G	874	60	^35^^,^^36^
	CTX-M-9-R	5′-TCA CAG CCC TTC GGC GAT			
	CTX-M-9_792_F	5′-CTA TTT TAC CCA GCC GCA AC	238	60	
	CTX-M-9_1029_r	5′-GTT ATG GAG CCA CGG TTG AT			

All amplified PCR products were exanimated by electrophoresis in a 1% Agarose, LE, Analytical Grade (Promega V3125 – USA) on 0.5X TAE Buffer (Promega V4281 – USA) and stained with SYBR™ Safe DNA Gel Stain (Invitrogen S33102 – USA). Amplicons’ size was estimated by 100 bp Plus DNA Ladder (Bioneer D-1035 – Korea). Gel casting and electrophoresis were performed in a horizontal electrophoresis system (CBS Scientific HSU-014, EPS-300X – USA). Specific bands were visualized on ENDURO™ UV Transilluminator (Labnet U1001 – USA). Raw and edited images of all PCR products are available in the *Underlying data.* ^37^ All betalactamese PCR products were sent for sequencing to Macrogen Inc. (Seul-South Korea), and obtained sequences were aligned against reference sequences by the web tool ResFinder 4.0. ^38^

### Statistical analysis

The free software R Studio (Version 1.4.1717) was used for statistical analysis. Descriptive statistics were used to facilitate the calculation of frequencies observed for each variable that would be used in the univariate analysis. For the bivariate analysis, we included variables of interest regarding the prevalence of *Salmonella* and then performed the chi-squared test and Fisher’s exact test. A univariate and multivariate logistic regression study was used to obtain odds ratios and 95% confidence intervals (CI). The level of significance was determined as *p* < 0.05.

## Results

The prevalence of *Salmonella* was 9.1% (15/165; CI _95%_ = 5.6-14.5).

### Serovar identification of *Salmonella*

The PCR technique allowed the identification of five *S.* Infantis and eight monophasic variants of *S.* Typhimurium (1,4,[5],12:i:-) isolates. Additionally, two *S.* Derby isolates were characterized by the Kauffman White scheme.

### Antimicrobial resistance analysis

Four antimicrobial resistance profiles were found in 11 isolates ( Table 4). Three and seven isolates of *S.* Infantis and *S.* 1,4,[5],12:i:- presented multi-resistant patterns, respectively. The two *S.* Derby and two *S.* Infantis isolates showed to be susceptible to all tested antibiotics.

**Table 4.** Antibiotic resistance patterns of *Salmonella* isolates *.

N° of antibiotic families	Resistance patterns	*S.* Infantis	*S.* 4,[5],12:i:-	Total
7	SAQBTFN	3	-	3
4	QBTF	-	1	1
3	QTF	-	6	6
2	TF	-	1	1
Total		3	8	11

S: Folate pathway inhibitor, A: Aminoglycosides, Q: Quinolones, B: Beta-lactams, T: Tetracycline, F: Phenicol, N: Nitrofurans. *S*. Derby and two *S*. Infantis isolates didn’t present resistance patterns.

* Four *Salmonella* isolates were susceptible to all tested antibiotics.

The highest resistance levels were present in three *S.* Infantis isolates with resistance phenotypes for 11 antibiotics. Additionally, all *S.* 4,[5],12:i:- isolates showed resistance to tetracycline and chloramphenicol ( Table 5). The *bla*_CTX-M-65_ gene was identified in all the *S.* Infantis isolates resistant to cefotaxime. On the other hand, none of the studied β-lactamase genes were identified in S. 4,[5],12:i:- isolates resistant to β-lactams.

**Table 5.** Resistance rates for tested antibiotics.

Antibiotic family	Antibiotic	Serotype	
		*S.* Infantis	*S.* 4,[5],12:i:-
Tetracycline	Tetracycline	60% (3)	100% (8)
	Tigecycline	0%	0%
Phenicol	Chloramphenicol	60% (3)	100% (8)
Quinolones	Ciprofloxacin	60% (3)	87.5% (7)
Beta-lactams	Ampicillin	60% (3)	12.5% (1)
	Cefotaxime	60% (3)	12.5% (1)
	Amoxicillin + Clavulanic acid	60% (3)	12.5% (1)
	Ceftazidime	40% (2)	0%
	Cefoxitin	40% (2)	0%
Folate pathway inhibitor	Sulfamethoxazole + Trimethoprim	60% (3)	0%
Aminoglycosides	Gentamicin	60% (3)	0%
	Amikacin	0%	0%
Nitrofurans	Nitrofurantoin	60% (3)	0%
Fosfomycin	Fosfomycin	0%	0%
Carbapenems	Ertapenem	0%	0%

*S*. Derby isolates were susceptible to all tested antibiotics.

### Risk factors for pork contamination

In bivariate analysis, the variable “Meat cutting surface wood” showed a significant association with *Salmonella* in pork ( *p* = 0.0349). Multiple logistic regression analysis determined that the variable “Meat preservation temperature” (higher than 8°C) also showed significant values ( *p* = 0.031) ( Table 6).

**Table 6.** Logistic regression model analysis of variables with significant values.

Variables	Standard error (SE)	*P* value	Odds Ratio	I.C. 95%	
Meat preservation temperature (>8°C)	0.1	0.03171 *	1.15	1.02	1.33
Meat cutting surface (wood)	1.6	0.01107 *	59.31	3.15	2 829.14

Full responses for the survey, metadata, and susceptibility test reports are available in the *Underlying data.* ^37^

## Discussion

This research aimed to determine the prevalence of *Salmonella* enterica in pork meat in public markets of Quito, Ecuador. Identification of *Salmonella* serovars, antimicrobial resistance profiles, and risk factors associated with the presence of the microorganism were included in this work.

In the present study, the prevalence of *Salmonella* in pork was 9.1% (95% CI = 5.6 – 14.5). Another study in Quito reported a higher *Salmonella* occurrence in pork in 2016. ^38^ This decrease in *Salmonella* occurrence could indicate that some interventions for improving market sanitary conditions could have been implemented. Several studies around the world have reported different levels of *Salmonella* in pork. In Europe, the prevalence of *Salmonella* in pork was 0.64% in 2019 (n=20 613), ^39^ while in Asian countries, the prevalence of S *almonella* varies from 14.1% to 57.74%. ^3^^,^^40^^,^^41^ Overall, variations of *Salmonella* prevalence within countries or among regions of the world could be associated with the implementation of specific technologies in meat treatment or the existence of specific regulations that could be put in place in Ecuadorian markets. ^42^ Nontheless, it must be mentioned that the epidemiology of *Salmonella* in raw meat is a multifactorial phenomenom and other local factors could influence the occurrence of this pathogen. ^5^^,^^11^

*Salmonella* Typhimurium monophasic variant ( *S.* 1,4,[5],12:i:-) was the predominant serovar in this study (53.4%), becoming the first report of this serovar in pig meat in Ecuador.

It has been mentioned that isolation of *S.* 1,4,[5],12:i:- has increased worldwide during the last 20 years. ^43^ Pigs are ranked as the main reservoir of this serovar in the European Union. ^44^ Furthermore, *S.* 1,4,[5],12:i:- has become one of the most common variants of *Salmonella* in pork production, ^45^^,^^46^ being considered an emerging serovar in many countries. ^46^ The present study demonstrates that this serovar may be relevant in the epidemiology of *Salmonella* linked to food contamination in Ecuador.

Concerning *S.* Infantis, our results differ from those reported by Mejia *et al.,* ^18^ who showed this serovar as the most isolated one in pig meat samples in retail markets in Ecuador. Since the later study samples were taken from supermarkets, this difference could be determined by the pig meat supply chain. In this regard, pork in traditional markets comes from many small pig production farms. On the other hand, pork from supermarkets comes from a few companies with intensive industrial production. These facts could explain the differences between studies. Moreover, *S.* Infantis has been reported as one of the most frequent serovars in industrial poultry production in many countries, ^47^^–^^49^ indicating that this serovar is well adapted to industrial conditions. ^50^^,^^51^ Since *S.* Infantis has been reported to cause human diseases, ^52^ the epidemiology of this serotype in pork and its relationship with other meat products (e.g., poultry) should be further investigated. The same criterion should be considered when approaching the epidemiological surveillance of S. Derby, since this *Salmonella* serovar has also been described as an important foodborne pathogen. ^11^^,^^53^

Antimicrobial resistance (AMR) of *Salmonella* isolates from pork and pig production has been reported in other countries at regional and global levels showing multi-resistant phenotypes. ^54^^,^^55^ However, AMR varies according to *Salmonella* serotypes. In this study, *S.* Infantis was the least susceptible serotype with resistance patterns of up to seven antimicrobial families. In the same way, *S.* Infantis isolates with high rates of AMR have been reported in the poultry industry of Ecuador, ^51^^,^^56^ which has been linked to the overuse of antimicrobials in this sector. ^57^ However, multi-resistant *S.* Infantis has been reported worldwide, demonstrating that multi-resistance is an important feature of this serovar, possibly linked to specific plasmids. Thus, the capacity of *S.* Infantis to acquire genetic determinants of resistance has been associated with the “plasmid of emerging *S. enterica* Infantis” (pESI) and related plasmids (pESI-like plasmids). ^58^ The *bla*_*CTX*-M-65_ gene identified in three *S.* Infantis isolates has been closely related to this megaplasmid in local studies ^51^^,^^56^^,^^59^^,^^60^ and globally. ^61^^–^^63^

The same can be said for *S.* 4, [5],12:i:- whose majority of isolates showed multi-resistant patterns. Moreover, this serotype has been reported in several countries, showing that *S.* 4,[5],12:i:- is specially adapted to the pig production sector and could represent a public health concern. ^64^^–^^66^

High levels of AMR *Salmonella* have been related to the incorrect use of antibiotics in animal production. The administration of cephalosporins and other beta-lactams in pig production has seen to increase in many countries, like France, ^67^ India, ^68^ and Brazil. ^69^ Moreover, in Ecuador, the misuse of antibiotics in animal production systems has been reported. ^70^^,^^71^ This aspect has particular relevance since beta-lactams are widely used in animal husbandry. ^72^^,^^73^ It must also be considered that ESBL genes could determine failure in treating diseased people with complicated infections caused by *Salmonella* and other bacteria to which horizontal transference of genes may occur. ^74^ In the present work, we found that meat preservation above 8°C (OR = 1.15) and wooden surfaces for meat cutting (OR = 59.31) were risk factors for *Salmonella* in pork meat. This is unsurprising since the lack of an appropriate cool chain allows the growth of contaminating microorganisms and pathogens in food products. ^75^ Besides, the porosity of wood in cutting tables will enable bacteria to grow in the presence of humidity, promoting cross-contamination of foodstuffs with *Salmonella.* ^76^^–^^78^ Whether *Salmonella* isolates recuperated in our study originated in the primary sector or are the result of such cross-contamination events should be further studied, targeting samples from pig production farms and conducting analysis of genomic clonality.

In conclusion, pork expended in traditional markets of Quito showed contamination of *Salmonella enterica,* whose main serovars are of public health concern. Beta-lactam-resistance in *Salmonella* isolates is also remarkable, which could become a risk for pork consumers.

## Data Availability

Figshare: PCR images of Prevalence, serovars, and risk factors associated with the presence of Salmonella in pork sold in public markets in Quito, Ecuador. https://doi.org/10.6084/m9.figshare.24195030.v1. ^37^ This project contains the following underlying data:
• CTX-M-9 Edited.jpg • CTX-M-9 RAW.jpeg • fljA-B-C edited.jpg • fljA-B-C raw.jpeg •
S_typhimurium_edited.jpg •
S_typhimurium_Raw.jpeg Figshare: Pork_database_risk_factors.xlsx. https://doi.org/10.6084/m9.figshare.23699505.v1. ^79^ Data are available under the terms of the Creative Commons Attribution 4.0 International license (CC-BY 4.0).
