# Supplementary material for: Prevalence, serovars, and risk factors associated with the presence of Salmonella in pork sold in public markets in Quito, Ecuador
Source: F1000Res. 2024 Jun 20;12:1367. Originally published 2023 Oct 18. [Version 3] doi: 10.12688/f1000research.138671.3 (PMC11176892; doi:10.12688/f1000research.138671.3)
Supplement: Peer review history

# Reviewer report: Reviewer response for version 3

**DOI:** 10.5256/f1000research.167981.r293433

**Published:** 2024-06-25

**Authors:** Ma Soledad Vázquez-Garcidueñas

**Affiliations:** 1 Facultad de Ciencias Médicas y Biológicas, Universidad Michoacana de San Nicolas de Hidalgo, Morelia, Michoacán, Mexico

All observations were attended to, just is only necessary to include in the PCR reactions the concentrations of the reagents used and since the amount of DNA cannot be entered, then the volume used must be entered. It is also necessary to include what equipment was used and the number of cycles.

Is the work clearly and accurately presented and does it cite the current literature?

Partly

If applicable, is the statistical analysis and its interpretation appropriate?

Yes

Are all the source data underlying the results available to ensure full reproducibility?

Yes

Is the study design appropriate and is the work technically sound?

Yes

Are the conclusions drawn adequately supported by the results?

Yes

Are sufficient details of methods and analysis provided to allow replication by others?

Yes

Reviewer Expertise:

Microbiology

I confirm that I have read this submission and believe that I have an appropriate level of expertise to confirm that it is of an acceptable scientific standard.

# Reviewer report: Reviewer response for version 2

**DOI:** 10.5256/f1000research.164525.r282788

**Published:** 2024-06-13

**Authors:** Xiaoping Liao

**Affiliations:** 1 South China Agricultural University, Guangzhou, China; 2 Guangdong Laboratory for Lingnan Modern Agriculture, Guangdong, China

In this study, the authors attempted to fill a data gap on the epidemiology, genomic characterization, and risk factor of *Salmonella *in Ecuador. Overall, the authors perform a detailed and comprehensive analysis of the strains and the manuscript is well-structured, well written and clear in its results and conclusions.

 I have several major concerns over the design and major findings of the study.

1. The term "multi-resistant *Salmonella *strains" has been used multiple times in the manuscript. Please replace it with "multidrug-resistant *Salmonella *strains."
2. Tables 2 and 3 should be relocated to the supplementary materials.
3. Regarding Table 3, please provide an explanation for the utilization of two pairs of primers in detecting the CTX-M 9 group.
4. The authors have inadvertently used "S. 1,4,[5],12:i:-" and "S. 4,[5],12:i:-" interchangeably in this manuscript. Please ensure consistency by using the same term throughout.

Is the work clearly and accurately presented and does it cite the current literature?

Yes

If applicable, is the statistical analysis and its interpretation appropriate?

Partly

Are all the source data underlying the results available to ensure full reproducibility?

Yes

Is the study design appropriate and is the work technically sound?

Yes

Are the conclusions drawn adequately supported by the results?

Yes

Are sufficient details of methods and analysis provided to allow replication by others?

Yes

Reviewer Expertise:

AMR

I confirm that I have read this submission and believe that I have an appropriate level of expertise to confirm that it is of an acceptable scientific standard, however I have significant reservations, as outlined above.

# Response

**Published:** 2024-06-14

**Authors:** Jose Luis Medina-Santana

**Affiliations:** Veterinary, Universidad Central del Ecuador, Quito, Pichincha, Ecuador

**Dear Xiaoping Liao,**

**South China Agricultural University, Guangzhou, China;  Guangdong Laboratory for Lingnan Modern Agriculture, Guangdong, China**

 Next, we will answer your questions and recommendations regarding the evaluated article.

 a) The term "multi-resistant Salmonella strains" has been used multiple times in the manuscript. Please replace it with "multidrug-resistant Salmonella strains."

**Response: changes are done.**

 b) Tables 2 and 3 should be relocated to the supplementary materials.

**Response: We think that these tables give important information for readers. So, we kept them in the manuscript.**

 c) Regarding Table 3, please provide an explanation for the utilization of two pairs of primers in detecting the CTX-M 9 group.

**Response: The *bla*_CTX_- _M 9 group_ is a big fragment to be sanger sequenced in only a reaction (around 1100pb). Additionally, several alleles integrate SNPs of differentiation in the terminal part of the sequence. So, the author’s primers designed two complementary pairs of primers to address this issue. More information can be found in the reference on the primers.**

 d) The authors have inadvertently used "S. 1,4,[5],12:i:-" and "S. 4,[5],12:i:-" interchangeably in this manuscript. Please ensure consistency by using the same term throughout.

**Response: Corrections are done.**

# Reviewer report: Reviewer response for version 1

**DOI:** 10.5256/f1000research.151889.r240450

**Published:** 2024-02-15

**Authors:** Ma Soledad Vázquez-Garcidueñas

**Affiliations:** 1 Facultad de Ciencias Médicas y Biológicas, Universidad Michoacana de San Nicolas de Hidalgo, Morelia, Michoacán, Mexico

This work is very relevant since it aimed to determine the prevalence, serovars, β-lactam resistance genes, and the risk factors associated with Salmonella enterica in pork since these animals are considered the reservoirs of many Salmonella serovars and strains related to antibiotic resistance, and this information is still limited in Ecuador. Identification of *Salmonella* serovars, antimicrobial resistance profiles, and risk factors associated with the presence of the microorganism were included in this work.

 It is recommended to make the following modifications:

 The conclusion of the summary is obvious and uninformative, it is recommended to write one that better describes the results.

 In the introduction, if the information in pork is limited in Ecuador, put a reference to how little is known or in any case change it to non-existent information.

 Since they amplified beta-lactamase, it is necessary to justify in the introduction why these were specifically sought.

 In materials and methods, change minutes and hours by min and h respectively.

 In PCR amplifications, include the amount of DNA contained in the assay.

 If possible, update the bibliography since there are some references that are too old.

Is the work clearly and accurately presented and does it cite the current literature?

Partly

If applicable, is the statistical analysis and its interpretation appropriate?

Yes

Are all the source data underlying the results available to ensure full reproducibility?

Yes

Is the study design appropriate and is the work technically sound?

Yes

Are the conclusions drawn adequately supported by the results?

Yes

Are sufficient details of methods and analysis provided to allow replication by others?

Yes

Reviewer Expertise:

Molecular epidemiology of Salmonella, Acinetobacter and Mycobacterium tuberculosis

I confirm that I have read this submission and believe that I have an appropriate level of expertise to confirm that it is of an acceptable scientific standard.

# Response

**Published:** 2024-04-04

**Authors:** Jose Luis Medina-Santana

**Affiliations:** Veterinary, Universidad Central del Ecuador, Quito, Pichincha, Ecuador

**Dear María Soledad Vázquez-Garcidueñas,**

**Facultad de Ciencias Médicas y Biológicas, Universidad Michoacana de San Nicolas de Hidalgo, Morelia, Michoacán, Mexico**

 Next, we will answer your questions and recommendations regarding the evaluated article.

 a) The conclusion of the summary is obvious and uninformative, it is recommended to write one that better describes the results.

**Response: **We changed conclusions in lines 30-33.

 b) In the introduction, if the information in pork is limited in Ecuador, put a reference to how little is known or in any case change it to non-existent information.

**Response: **The reference is added in line 67.

 c) Since they amplified beta-lactamase, it is necessary to justify in the introduction why these were specifically sought.

**Response: **The reference is mentioned in line 64.

 d) In materials and methods, change minutes and hours by min and h respectively.

**Response: **words have been changed.

 e) In PCR amplifications, include the amount of DNA contained in the assay.

**Response:** We used the boiling method as described. We did not perform DNA quantification since normally this method provides enough free DNA for PCR amplification.

 f) If possible, update the bibliography since there are some references that are too old.

**Response: **References have been updated according to the commentaries of reviewer 1.

# Reviewer report: Reviewer response for version 1

**DOI:** 10.5256/f1000research.151889.r234636

**Published:** 2024-02-15

**Authors:** Patricio Retamal

**Affiliations:** 1 Departamento de Medicina Preventiva Animal, Facultad de Ciencias Veterinarias y Pecuarias, Universidad de Chile, Santiago, Chile

This article describes the detection of Salmonella serovars in pork meat from several markets in Quito, Ecuador. It's an interesting work that depicts contamination rates of this food commodity and raises concerns about public health risks for people involved in processing or consuming this product due to the presence of multidrug-resistant pathogenic bacteria.

 However, there are some major comments about the text that should be addressed by the authors before indexing.

 A major issue is related to the serotyping methodology. The PCR reaction for serotyping should be backed up with the traditional Kauffmann-White scheme or with whole-genome sequencing. Because PCR may have some false positive results (as indicated in the cited reference), all isolates should have been confirmed with KW. Why did the authors do it partially?

 Conclusions, both in the abstract and within the main text, should address the main findings of this work.

 Cited references need an update. Just one of them is from 2023, and some data is definitely old. For instance, the first paragraph of the introduction indicates cases from 2010.

 The text should be revised by a native English speaker. There are several mistakes throughout the manuscript.

 What was the criterion for the selection of markets? Clarification is required.

 What was the criterion for the selection of butcheries and interviewed employees?

 Normally, variables included in epidemiological surveys are selected from previous reports or locally identified during in situ observations. What were the sources for the included variables?

 Other minor comments:

 The Kauffmann-White serotyping is a standard procedure that does not require a detailed description. A reference is enough. The same occurs with the Kirby-Bauer method.

 What was the control strain in the Kirby-Bauer procedure? It is required to validate results.

 Tables 4 and 5 should be merged, and optimally, all isolates should be described (antimicrobial phenotypes and genotypes) in this new table.

 In the discussion, the second paragraph, the potential causes of differences between Salmonella prevalence values between countries or continents are multifactorial, but authors identify "technologies for meat treatment or specific regulations." I think a broader analysis should be made for observed differences.

 In the discussion, paragraphs 3 and 4 should be merged.

 In the discussion, the fifth paragraph, I don't understand the statement "because the surveillance of Salmonella serovars shapes the epidemiology of these pathogens in foodstuffs." Please give some clarification.

 Please verify references 6 and 8. I think they are incompletely written.

Is the work clearly and accurately presented and does it cite the current literature?

Partly

If applicable, is the statistical analysis and its interpretation appropriate?

Yes

Are all the source data underlying the results available to ensure full reproducibility?

Yes

Is the study design appropriate and is the work technically sound?

Partly

Are the conclusions drawn adequately supported by the results?

Partly

Are sufficient details of methods and analysis provided to allow replication by others?

Yes

Reviewer Expertise:

Microbiology

I confirm that I have read this submission and believe that I have an appropriate level of expertise to confirm that it is of an acceptable scientific standard, however I have significant reservations, as outlined above.

# Response

**Published:** 2024-04-04

**Authors:** Jose Luis Medina-Santana

**Affiliations:** Veterinary, Universidad Central del Ecuador, Quito, Pichincha, Ecuador

**Dear Patricio Retamal** **,**

**Departamento de Medicina Preventiva Animal, Facultad de Ciencias Veterinarias y Pecuarias, Universidad de Chile, Santiago, Chile**

 Next, we will answer your questions and recommendations regarding the evaluated article.

 a) A major issue is related to the serotyping methodology. The PCR reaction for serotyping should be backed up with the traditional Kauffmann-White scheme or with whole-genome sequencing. Because PCR may have some false positive results (as indicated in the cited reference), all isolates should have been confirmed with KW. Why did the authors do it partially?

**Response:**

 PCR used in this research has been extensively proved as an appropriate method for the tested serotypes in this research (Infantis, Enteritidis, Typhimurium, and (1,4, [5], 12: i:-)). Moreover, we have proved its specificity compared with WGS in more than 800 *Salmonella* isolates of our collection. Therefore, we think that it is a reliable method to access the serotype of *Salmonella* isolates of this study. In Ecuador, there is not a reference laboratory for *Salmonella* serotyping. Therefore, performing KW in all isolates is cumbersome and costly and was not included in this project. It is important to mention that WGS was later performed in these isolates confirming the serotyping results. Information of WGS is not included in this manuscript since it is part of another publication.

 b) Conclusions, both in the abstract and within the main text, should address the main findings of this work.

**Response:** We changed conclusions in lines 30-33 **.**

 c) The cited references need an update. Just one of them is from 2023, and some data is definitely old. For instance, the first paragraph of the introduction indicates cases from 2010.

**Response: **We updated the references in lines 41-43 and 45-47. References for other parts of the manuscript are updated.

 d) The text should be revised by a native English speaker. There are several mistakes throughout the manuscript.

**Response: **The text has been revised.

 e) What was the criterion for the selection of markets? Clarification is required. What was the criterion for the selection of butcheries and interviewed employees?

**Response: **Clarification added in 73 - 78

 f) Normally, variables included in epidemiological surveys are selected from previous reports or locally identified during in situ observations. What were the sources for the included variables?

**Response: **Clarification added in 84 - 85

 g) The Kauffmann-White serotyping is a standard procedure that does not require a detailed description. A reference is enough. The same occurs with the Kirby-Bauer method.

**Response: **The Kauffmann-White method has been deleted and replaced in lines 135 to 137 according to the previous commentary. Reference to the Kirby-Bauer method is included in line 137 and additional text has been deleted.

 h) What was the control strain in the Kirby-Bauer procedure? It is required to validate the results.

**Response: **information added in lines 152-153.

 i) Tables 4 and 5 should be merged, and optimally, all isolates should be described (antimicrobial phenotypes and genotypes) in this new table.

**Response: **tables 4 and 5 show different data. We think they should be displayed separately for a better understanding. We do not report genotypes in this study.

 j) In the discussion, the second paragraph, the potential causes of differences between Salmonella prevalence values between countries or continents are multifactorial, but authors identify "technologies for meat treatment or specific regulations." I think a broader analysis should be made for observed differences.

**Response: **Clarification added in 225 - 227

 k) In the discussion, paragraphs 3 and 4 should be merged.

**Response: **Paragraphs merged.

 l) In the discussion, the fifth paragraph, I don't understand the statement "because the surveillance of Salmonella serovars shapes the epidemiology of these pathogens in foodstuffs." Please give some clarification.

**Response:** Clarification added in 247-249.

 m) Please verify references 6 and 8. I think they are incompletely written.

**Response**: references verified.
